# Principles and Practice of Epidemiology Avoid Statistical Esotericism

**Published:** 2005-06

**Authors:** Reimert T. Ravenholt

**Affiliations:** *Population Health Imperatives, Seattle, Washington, USA*

## BODY

In previous papers, I have stated that routine presentation of tests of the statistical significance of numerical findings in reports of epidemiological studies, and routine presentation of Confidence Intervals accompanying the numbers presented in such reports, is an unfortunate and invalid practice: because such extra precise analysis of numerical study results - while neglecting analogous precise measurement of non numerical determinants of study results - violates the basic scientific Consistent Precision Principle (CPP) (1, 2). Highly experienced epidemiological colleagues have communicated their hearty agreement with this view; while some younger colleagues have indicated their habitual dependence upon such Confidence Interval “crutches” when reading epidemiological reports.

Now I wish to further state that such statistical esoterica have been inordinately used by many statisticians in their self appointed role as “sidewalk superintendents” of epidemiological studies: as reins with which they could “mount and ride” epidemiological studies done by others; thereby avoiding much of the tedium of planning and implementing their own epidemiologic studies while yet maintaining their Ivory Tower existence. Far better if they emulate the superb epidemiological studies done and well analyzed by statistical lions such as Raymond Pearl (3) and E. Cuyler Hammond (4).

Excessive interjection and concern for statistical esoterica in the analysis of numerical findings diverts attention from the patterns of epidemiological findings often essential for understanding the meaning of study findings. Chi Square tests are notoriously incapable of definitive discernment of data patterns (5). For thorough consideration of data patterns, the data should be presented in grids bordered by three foremost known determinants, time, sex, and age (6), while observing successively the distribution of cases or deaths from diseases of interest, according to race, wealth, religion, etc; thus enabling the researcher to perceive and understand the interactions between foremost determinants of the phenomenon being studied. Such stratified sequential analyses of study data, with appropriate charting, enables researchers to gain an intimate and more powerful understanding of research findings and meanings. Also, with the aid of computers it is now readily possible to perform multivariate analyses, controlling for many known or suspected determinants while studying the operation of one or more selected putative determinants (7). But this greater data handling capability is not infrequently attended by errors which invalidate the results. A case in point is the multivariate studies ostensibly giving due weight to the smoking experience of study subjects while considering the effects of many lesser determinants of disease and death, e.g. diet and activity, but using such grossly inadequate measures of lifetime smoking experience that the ex smoker category lumps subjects who have smoked only 100 cigarettes with those who have smoked more than 500,000 cigarettes, thus minimizing the apparent pathogenic effects of tobacco while enabling the investigators to magnify the effects of their favored alternative putative determinants. When ill defined elements are included in a multivariate analysis, acceptance of the result as meaningful becomes an act of faith rather than science. In the case of smoking experience, lifetime exposure should be more adequately measured by charting average daily consumption of cigarettes, pipefuls or cigars by year of age, and converting the areas under charted lines to the approximate number of lifetime smoking exposure units (8).

Although outlier data constitute less than 5% of all data, they may yet be of compelling importance. Careful attention to outliers is needed for sound interpretation of data findings somewhat as a wise sheepherder seeking to know where his flock is going, judges both the position and movement of the main flock and the position and movement of fringe sheep (outliers).

Use of statistical esoterica to refine the findings and meanings of case-control studies is especially ludicrous - because of the inescapable uncertainties interjected into such studies by the inherently crude comparability of the cases and controls (9).

Habitual reliance upon *p*-values and Confidence Intervals when analyzing epidemiologic reports, misdirects the attention of readers to consider as significant and worthy of credence only those values which fall within the orthodox 95% confidence intervals. This is most unfortunate. Because the purported gain from avoidance of alpha errors is canceled by the inescapable increase in beta errors. Wise men and women would not entrust epidemiological leadership to researchers whose judgment of what is significant is limited to 95% of study results: because the 5% of findings lying outside their purview not infrequently contains information vital for solution of difficult epidemiologic puzzles.

Statistical esoterica have gained false credence as important tools for routine analysis of study data because of the mistaken belief that the purpose of a single epidemiological study is to prove a demonstrable relationship. This is not its legitimate purpose; it is beyond the capability of a single study to prove anything - no matter what statistical esoterica are employed because of the inherent inescapable crudeness of many non numerical determinants of study findings, especially the skill and dependability of all key researchers contributing to the study: thus dictating that all that can reasonably be expected from a single epidemiological study (no matter how excellent) is that it point the way - that it establish a new paradigm for other researchers. Only by combining the findings of many researchers and by understanding the operative mechanisms, does one gain a sound basis for firm belief in study results. Hence, insertion of p values and Confidence Intervals into epidemiological reports is as useless and harmful as if inserted into the Wall Street Journal or the New York Times.

The approximate reliability of numerical study results, when the actual data are presented without CIs, is readily judged by experienced researchers armed by well taught elementary probability courses and substantial epidemiologic experience. Whereas, the addition of a blizzard of accompanying Confidence Intervals forces the reader to either devote considerable extra time to reading each CI and judging the more complex data set, or - as most readers do - passing over the more complex data set lightly while assuming the data are reliable because the author has calculated all those Confidence Intervals - now computed by a few flicks of a finger. Most nonsensical of current numerical practices is the presentation of naked percentages bolstered with Confidence Intervals, instead of the traditional presentation of the operative numbers with accompanying percentages. Presentation of naked percentages rather than the operative values was a common Russian totalitarian practice when seeking to conceal the actual sorry state of the Russian economy; preventing critical researchers from readily combining and analyzing findings from multiple studies and nations.

Whether listing double numbers indicating Confidence Intervals, e.g. 530.35 (524.68-535.98), or a plus-minus number indicating a 95% Confidence Interval, e.g. 1085, 84.2%, both practices clutter the data pages unnecessarily and fail the utility test when compared with the traditional practice of simply presenting the sample size, the number of events observed in that sample, and the percent of the sample size manifesting the events being studied. Able epidemiologists and statisticians during centuries gained adequate understanding of the approximate meaning and stability of percentages generated by stated numbers without cluttering their articles with innumerable Confidence Intervals. The cluttering of epidemiological journals with Confidence Intervals during the last several decades is an invalid attempt by a new generation of neophyte epidemiologists to negotiate the shoals of epidemiological practice from Ivory Towers without gaining the shoe-leather epidemiological experience/expertise characteristic of leading epidemiologists.

Neophyte epidemiologists reading articles replete with numerous 95% Confidence Intervals, ostensibly guarding against misinterpretation of the findings presented, are misled to believe that composite study conclusions are thereby likewise guarded by a 95% Confidence Interval. But that this is not so, is readily demonstrated simply by multiplying the Confidence Interval by itself numerous times: 0.95 × 0.95 × 0.95 × 0.95 × 0.95 × 0.95 × 0.95 × 0.95 × 0.95 × 0.95 × 0.95 × 0.95 × 0.95 × 0.95 = 0.49. Hence, readers should be especially wary of accepting author conclusions when reading reports containing a blizzard of Confidence Intervals - often aimed mainly at obscuring the fact that non numerical determinants of the study results are seriously flawed.

Truly, the Statistical Esoterica Emperor has no clothes on.
